# DNA Demethylation in Response to Heat Stress in *Arabidopsis thaliana*

**DOI:** 10.3390/ijms22041555

**Published:** 2021-02-04

**Authors:** Urszula Korotko, Karolina Chwiałkowska, Izabela Sańko-Sawczenko, Miroslaw Kwasniewski

**Affiliations:** 1 Centre for Bioinformatics and Data Analysis, Medical University of Bialystok, 15-089 Bialystok, Poland; urszula.korotko@umb.edu.pl (U.K.); karolina.chwialkowska@umb.edu.pl (K.C.); 2 Department of Genetics, University of Silesia in Katowice, 40-007 Katowice, Poland; 3 Department of Botany, Institute of Biology, Warsaw University of Life Sciences, 02-787 Warszawa, Poland; izabela_sanko_sawczenko@sggw.pl

**Keywords:** abiotic stress, epigenetics, heat shock proteins

## Abstract

Environmental stress is one of the most important factors affecting plant growth and development. Recent studies have shown that epigenetic mechanisms, such as DNA methylation, play a key role in adapting plants to stress conditions. Here, we analyzed the dynamics of changes in the level of DNA methylation in *Arabidopsis thaliana* (L.) Heynh. (Brassicaceae) under the influence of heat stress. For this purpose, whole-genome sequencing of sodium bisulfite-treated DNA was performed. The analysis was performed at seven time points, taking into account the control conditions, heat stress, and recovery to control conditions after the stress treatment was discontinued. In our study we observed decrease in the level of DNA methylation under the influence of heat stress, especially after returning to control conditions. Analysis of the gene ontology enrichment and regulatory pathways showed that genes characterized by differential DNA methylation are mainly associated with stress response, including heat stress. These are the genes encoding heat shock proteins and genes associated with translation regulation. A decrease in the level of DNA methylation in such specific sites suggests that under the influence of heat stress we observe active demethylation phenomenon rather than passive demethylation, which is not locus specific.

## 1. Introduction

Environmental stress is one of the most important factors affecting the growth and development of plants [1]. Due to the fact that plants are continuously exposed to adverse environmental conditions, they have developed a variety of mechanisms that enable them to survive in stress conditions [2]. These mechanisms operate at the physiological and biochemical level [3]. Plant cells receive stress signals through various sensors and the signals are transduced by multiple signaling pathways [4]. Many second messengers, plant hormones, signal transducers and transcriptional regulators are involved in these pathways [5]. One of the most important stress factors for plants is high temperature [6]. Plant response to heat stress is a complicated process in which the main role is played by heat shock proteins (HSPs). HSPs act as molecular chaperones to prevent denaturation or aggregation of target proteins as well as facilitating protein refolding [7]. However, numerous biochemical and metabolic traits are also involved in the development and maintenance of thermotolerance. Examples are antioxidant activity, membrane lipid unsaturation, gene expression and translation, protein stability, and accumulation of compatible solutes [8]. So far, the role of many proteins in response to heat stress have been confirmed. One example is late embryogenesis abundant (LEA) proteins, which can prevent aggregation and protect the citrate synthase (involved in ATP production) from desiccating conditions like heat and drought stress [9]. Similarly, ubiquitin and conjugated-ubiquitin synthesis emerged as an important mechanism of heat tolerance in mesquite and soybean experiencing heat stress [10]. Another example is the chloroplast protein synthesis elongation factor (EF-Tu), which has a proven role in plant response to high temperature stress [5,11,12,13].

Recent studies have shown that epigenetic mechanisms are crucial in plant adaptation to stressful conditions [14]. Genetic and epigenetic regulation, which play important role in abiotic stress gene networks, include changes in histone modification, DNA methylation, and non-coding RNAs. DNA modifications, such as methylation of cytosine, and histone modifications can modulate the chromatin structure and thereby alter the accessibility of particular regions of the genome for transcription complex and therefore can affect genes expression level [15]. Understanding how it comes to changes in DNA methylation under stressful conditions, such as high temperature, will allow a better understanding of the mechanisms of plant response to environmental factors.

Unlike animals, for which DNA methylation mainly occurs in a CpG context, plant methylomes also encompass non-CpG methylation in CHG and CHH contexts (where H represents any nucleotide other than G) [16]. Plants possess at least three mechanisms of DNA methylation that differ based on sequence contexts [17]. The CpG sites are maintained by DNA METHYLTRANSFERASE1 (MET1) and CHROMOMETHYLASE1 (CMT1), while the CHG sites are maintained by CHROMOMETHYLASE3 (CMT3) however, CHH sites are maintained by constant *de novo* methylation by DOMAINS REARRANGED METHYLTRANSFERASE2 (DRM2) and other players in the RNA-directed DNA methylation (RdDM) pathway [18]. DNA demethylation can be carried out in two ways, passive and active [19]. Passive DNA demethylation is caused by lack of DNA methyltransferase activity or shortage of a methyl donor following DNA replication which results in failure to maintain methylation [20]. Active DNA demethylation requires a team of enzymes. In plants, a family of bifunctional 5-mC DNA glycosylases–apurinic/apyrimidinic lyases initiates active DNA demethylation through a base excision repair pathway [21]. *Arabidopsis thaliana* (L.) Heynh. (Brassicaceae) has a family of four bifunctional 5-mC DNA glycosylases, including REPRESSOR OF SILENCING 1 (ROS1), TRANSCRIPTIONAL ACTIVATOR DEMETER (DME), DEMETER-LIKE PROTEIN 2 (DML2) and DML3, which can excise 5-mC from all cytosine sequence contexts [21,22,23,24].

Previous studies provide examples of how DNA methylation affects plants tolerance to abiotic and biotic stress factors. It has been demonstrated that DNA methylation has a role in response to drought and salinity stress in rice [25]. In these studies, three rice cultivars (IR64, Nagina22, Pokkali) with contrasting response to drought and salinity stress, were used to reveal the epigenetic regulation of abiotic stress response. Significant differences were observed in the methylation patterns in different rice cultivars with contrasting stress response. In another study [26] authors, using *A. thaliana* mutants of DNA methyltransferases, histone-modifying enzymes, chromatin remodelers, and genes involved in small RNA biogenesis, provided evidence that the transcriptional response to temperature stress, at least partially, relies on the integrity of the RNA-directed DNA methylation pathway. Other research showed the role of DNA methylation in regulation of the *A. thaliana* immune system [27]. In these studies, authors showed that DNA methylation imparts control over the *A. thaliana* defense response against the biotrophic pathogen *Pseudomonas syringae pv. tomato DC3000* (*Pst*). Detailed studies showed that some defense genes are modulated by DNA methylation, as mutants globally lacking CG or non-CG methylation display constitutive and inducible misexpression of pathogen-responsive genes. These transcriptional alterations correlate with a dramatic enhanced Pst resistance phenotype in the *met1-3* and *ddc* mutant plants.

In the present work, we show dynamics of genome-wide DNA methylation level during heat stress treatment and after recovery to control conditions. We indicated global patterns of DNA methylation and identified specific regions of genome in which changes in DNA methylation occur under the influence of heat stress. We have also described examples of genes undergoing DNA methylation regulation under heat stress and we have shown that they are closely related to stress response. This study is the first such detailed analyses of the changes in DNA methylation level in *A. thaliana* under heat stress at global scale and at many time points, also after treatment discontinuation and transfer to control conditions.

## 2. Results

### 2.1. Experiment Design and Whole-Genome Bisulfite Sequencing

**Figure 1:**
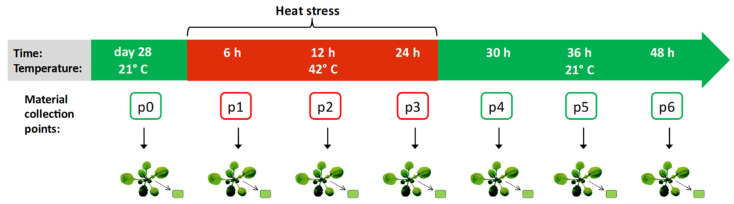
Scheme of the experiment. Samples of *Arabidopsis thaliana* were collected in seven time points: Under control conditions (marked as p0), during heat stress (p1, p2, p3), and after returning to the control conditions (p4, p5, p6) with three replicates for each time point.

To analyze the dynamics of DNA methylation during and after heat stress in A. thaliana, whole-genome sequencing was performed on bisulfite-treated DNA extracted from leaves of wild-type A. thaliana seedlings subjected to high temperature. The temperature and treatment duration were selected based on the literature [26,28,29] and preliminary experiments. The assumption was to treat plants with the highest possible stress that do not cause plant death. Based on the conducted analyzes, we set the temperature at 42 °C and the treatment time of 24 h. Samples were collected at seven time points: Under control conditions, during heat stress (after 6 h, 12 h and 24 h), and after returning to the control conditions (after 6 h, 12 h, and 24 h) with three replicates for each time point. Experiment scheme is presented in Figure 1. The reads sequenced from the control and high temperature-treated plants were mapped onto the A. thaliana genome with an efficiency of 70% on average, which resulted in 18–27 million paired-end alignments with a unique best hit and 410–660 million cytosines in each sample.

### 2.2. Heat Stress induces DNA Demethylation in Genes

In order to analyze the dynamics of changes in the level of DNA methylation under the influence of heat stress, we conducted differential methylation analysis. The aim of the analysis was to find out where changes in the level of DNA methylation occur in the genome, how quickly changes in the level of DNA methylation occur under influence of heat stress, and what is the direction of these changes, i.e., whether the level of DNA methylation increases or decreases. Using beta-binomial model implemented in Radmeth [30] we identified differentially methylated cytosines (DMCs) in all three sequence contexts. We compared pairwise samples from all time points to track changes in the subsequent stages of the experiment. This analysis was carried out separately for each of the CpG, CHG, and CHH methylation contexts. Differentially methylated cytosines with *p* < 0.01 after false discovery rate correction were treated as significant.

**Figure 2:**
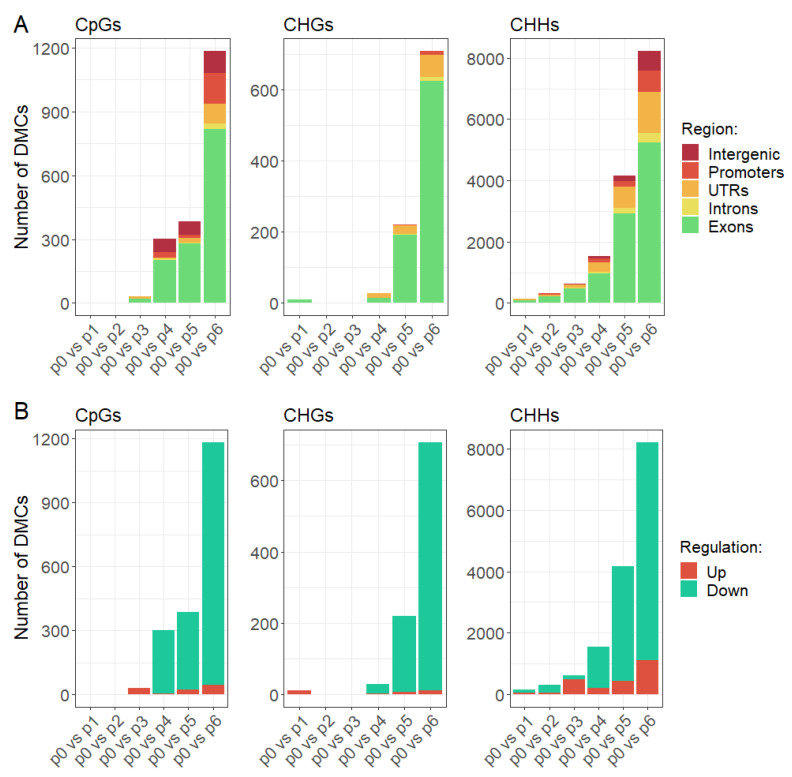
Differentially methylated cytosines in CpGs, CHGs, and CHHs in subsequent time points in comparison to control sample depending on genomic region (**A**) and direction of change (**B**).

The first stage of the analysis was to check how many hours after the heat stress treatment the DNA methylation level changed. By comparing successive time points to the control samples, we observed a delay in the appearance of changes in DNA methylation level, as most of the changes we identified did not occur until the heat stress treatment was discontinued and the plants returned to control conditions (Figure 2). Most of the changes in the level of DNA methylation that we observed occurred 24 h after the end of the heat stress treatment. A very small part of the identified differentially methylated cytosines occurred in the initial stages of the experiment, that is during high temperature treatment. In particular, this applies to cytosines in the CpG and CHG context, where almost no changes in the level of DNA methylation were observed during stress treatment.

In order to check in which genomic regions changes in the level of DNA methylation occurred, we annotated differentially methylated cytosines and assigned them to categories such as exons, introns, UTRs, promoters, and intergenic regions. Our analysis revealed that a significant part of all identified DMCs was found in the genes, in particular in the coding part, while relatively few DMCs have been identified in the intergenic regions (Figure 2A), especially in the case of CHGs, where we observed changes in DNA methylation level almost exclusively in exons and UTRs. Cytosines in exons covered by our experiment accounts for 30 to 40% of all analyzed cytosines depending on the cytosine context, while differentially methylated cytosines in exons constitute 60–80% of all identified DMCs depending on the cytosine context. In turn, cytosines in intergenic regions accounts for 24 to 29% of all analyzed cytosines depending on the cytosine context, while differentially methylated cytosines in intergenic regions constitute 1–8% of all identified DMCs depending on the cytosine context. Therefore, given such over-representation of DMCs in exons and under-representation in intergenic regions we can conclude that changes in the level of DNA methylation concern primarily coding regions.

In the next step we checked the direction of changes in the methylation level of the identified DMCs. We observed that the majority of identified differences in DNA methylation pattern concern the reduction of the methylation level (Figure 2B). A very small proportion of the differentially methylated cytosines showed an increase in the level of DNA methylation. Interestingly, changes in DNA methylation level observed in the early stages of the experiment concerned mainly the increase in DNA methylation. The results suggest the occurrence of two phenomena. The first is the increase in the level of DNA methylation during heat stress in a small portion of the genome. The second, much more frequent, is the reduction of DNA methylation level after the end of stress treatment and transfer to the control conditions. The same observation applies to cytosines in all contexts.

As the most of the differentially methylated cytosines in all sequence contexts were identified in genes, we decided to conduct further analysis focusing primarily on gene sequences. As the most interesting sites are those where DNA methylation changes occur in a larger area, we wanted to find genes with differentially methylated regions. We identified differentially methylated genes using predefined regions containing information about exons, introns, and UTRs of all A. thaliana genes. Our analysis allowed us to identify genes in which DNA methylation level has changed in the entire gene body or only in its part, for example, in one of the exons. Differentially methylated region was defined as a region with at least five differentially methylated cytosines and q-value < 0.05. The same as in the case of differentially methylated cytosines, we compared pairwise samples from all time points to track changes in the subsequent stages of the experiment. Taking into account all comparisons, we identified 123 differentially methylated genes with differences in methylation level in CpGs, 180 with differences in methylation level in CHGs and 724 with differences in methylation level in CHHs (Table S1). Almost all identified differentially methylated regions were located in coding part of genes, with no differences in introns and a few cases in UTRs. As in the case of the DMCs analysis, most of the changes in the level of methylation that we observed occurred at the final stages of the experiment, that is during recovery to optimal conditions.

**Figure 3:**
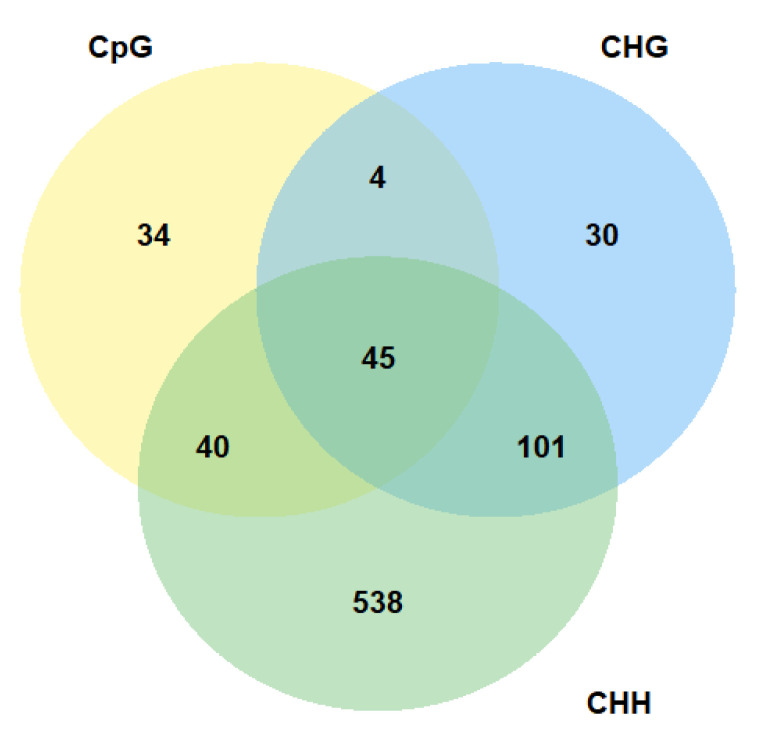
Number of differentially methylated *Arabidopsis thaliana* genes in different sequence contexts.

**Figure 4:**
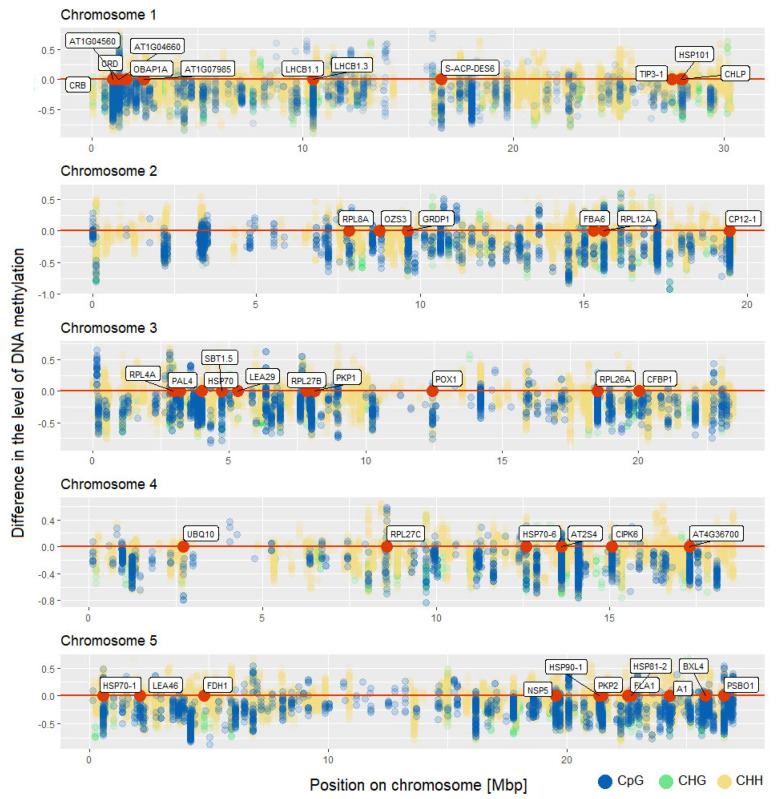
Location of identified differentially methylated cytosines in all comparisons in *Arabidopsis thaliana* genome in CpG, CHG, and CHH contexts. The y-axis shows the difference in the level of DNA methylation. Location of genes that are differentially methylated in all sequence contexts is shown as red dots.

The majority of genes with altered methylation level in CpG context were also differentially methylated in other cytosine context (Figure 3). Above 35% of all genes with altered methylation level in CpG context were differentially methylated in all cytosine contexts. We identified 45 such genes. A similar number of genes were common for CpG and CHH context (40 genes). However, there is also a large group of genes that has altered methylation level only in CHHs. We identified 538 such genes. The location of genes that are differentially methylated in all sequence contexts along with location of all identified differentially methylated cytosines in all comparisons in CpG, CHG, and CHH contexts is presented in Figure 4. Our analysis shows that the identified changes in DNA methylation level are not distributed randomly in the genome.

**Figure 5:**
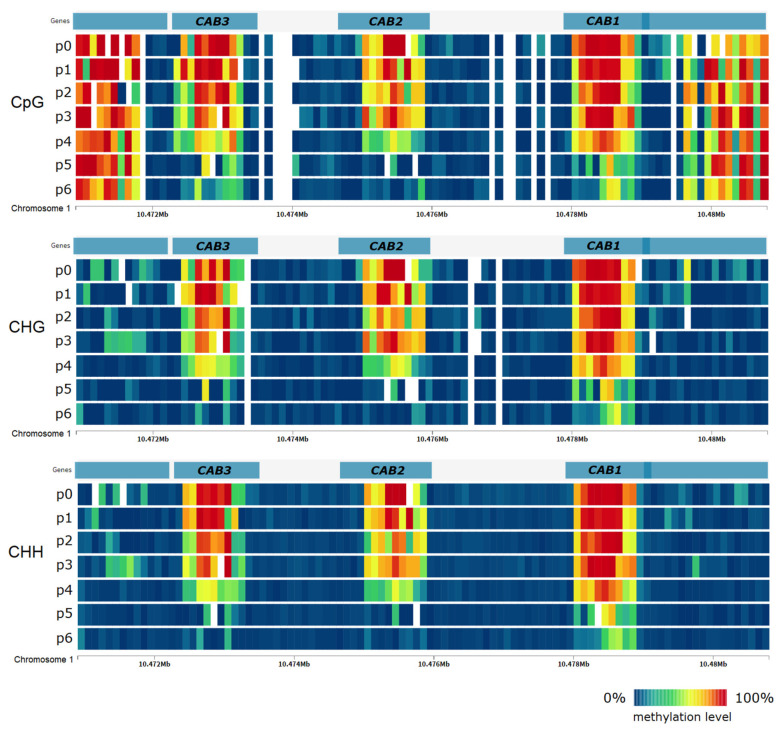
DNA methylation level in *Arabidopsis thaliana* genes encoding chlorophyll a-b binding proteins (CAB1, CAB2, CAB3) under heat stress in the subsequent time points of the experiment. DNA methylation level does not change significantly during heat stress (p1–p3) but decreases after transfer to normal conditions (p4–p6).

Taking into account the direction of changes, that is to say, whether there is a decrease or increase in DNA methylation level, we observed a similar phenomenon as in the analysis of single cytosines, namely, decrease in DNA methylation in most genes, a few hours after discontinuing treatment of heat stress. DNA methylation level of these genes does not change significantly under the influence of stress, but decreases after discontinuation of stress in the last points of the experiment. Often, changes in DNA methylation levels occur across all three sequence contexts as in the case of genes encoding chlorophyll a-b binding proteins (AT1G29910, AT1G29920, AT1G29930) shown in Figure 5. However, in the case of several genes, we observed an increase in DNA methylation level during heat stress treatment and then a decrease after its discontinuation and transfer to control conditions. We have not identified any gene in which DNA methylation level increases under stress and does not decrease after treatment discontinuation and transfer to control conditions.

As the gene-associated DNA methylation changes can occur within the gene body or in the promoter [20], we were also interested in identification of promoters with differentially methylated regions. We identified differentially methylated promoters using predefined regions containing information about location of promoters of each of the A. thaliana genes. Promoter was defined as a region up to 1000 bp long upstream from transcription start site, however promoter region might be shorter than 1000 bp if there are other genes in this range. We find only eight genes with differentially methylated promoters and only in CpG context, but it is not extraordinary due the fact that in A. thaliana, only approximately 5% of the genes are methylated in promoter regions and DNA methylation does not regulate the transcription of many genes [20]. All the changes identified concerned a decrease in the level of DNA methylation. The majority of genes with differentially methylated promoters identified by us are hypothetical proteins or non-coding RNAs without information about their function.

### 2.3. Genes Undergoing DNA Demethylation under Heat Stress are Associated with the Stress Response

**Figure 6:**
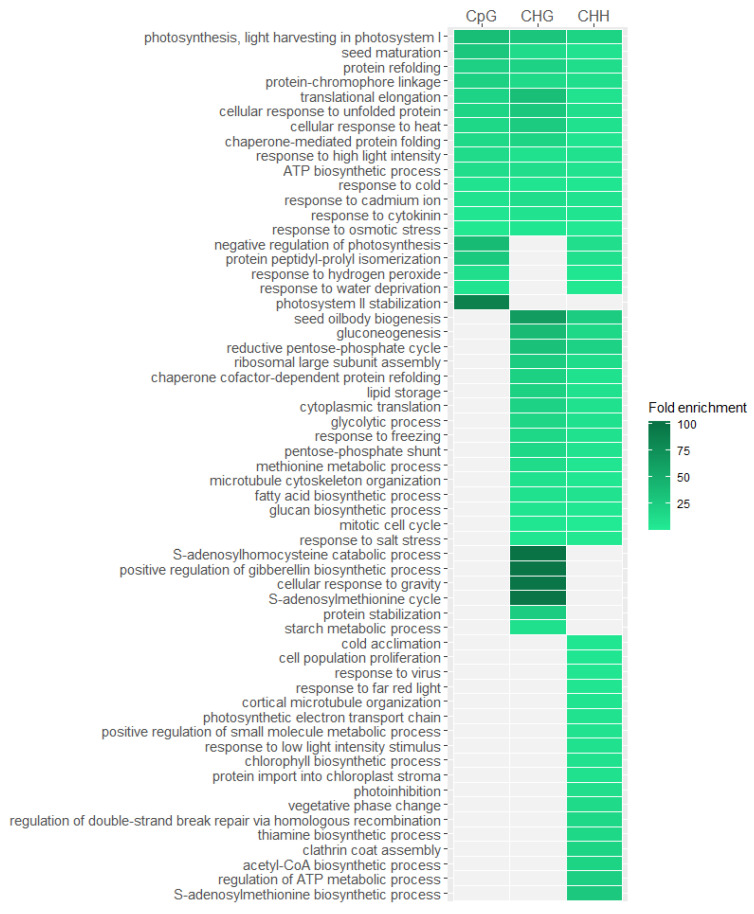
Biological processes overrepresented in differentially methylated *Arabidopsis thaliana* genes in different sequence contexts (CpG, CHG, CHH). Only the most significant processes from the gene ontology hierarchy tree are presented.

To check whether identified changes in DNA methylation pattern are stress-specific and emerged under the influence of heat stress treatment, we conducted ontology enrichment analysis of differentially methylated genes. Due to the fact that we have identified very few genes that methylation level increased under heat stress and those that react at the initial stages of the experiment, we took into account all genes that methylation profile changed under the influence of stress. We conducted the analysis separately for genes with differences in CpGs, CHGs and CHHs (Figure 6). Results with a significance of *p* < 0.05 after false discovery rate correction were taken to be significant. Our analysis indicated that overrepresented features are mainly related to the stress response, especially those that are common to genes with changed methylation profile in all three sequence contexts. Among these features we can mention those associated with protein refolding which is a process strictly related to the response to heat stress and regulated by heat-shock proteins. Other interesting overrepresented features is translational elongation that is a process highly affected by heat stress according to literature [31]. We also observe many overrepresented features related to response to various stimuli like osmotic stress, heat, cold, metal ion, and high light intensity. Another overrepresented feature is seed maturation, which may be explained by the fact that many genes involved in seed maturation are also responsible for stress response like for example genes belonging to LATE EMBRYOGENESIS ABUNDANT (LEA) family [32] or genes associated with abscisic acid [33]. The results suggest that very specific genes related with stress response are subjected to DNA methylation changes under high temperature treatment. However, we have observed that genes in which changes in DNA methylation level have only occurred in the CHG or CHH context but not in CpG are less related to the heat stress response, suggesting that the changes in methylation level in this sequence contexts are less specific.

**Figure 7:**
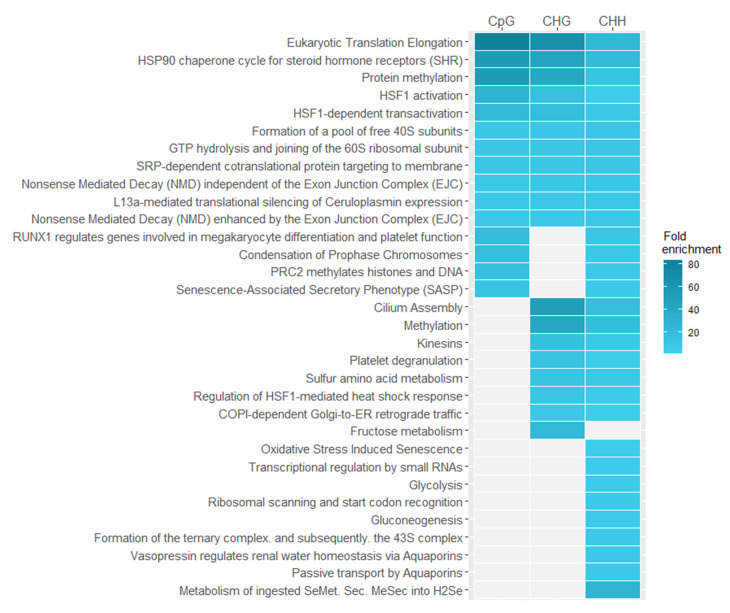
Pathways overrepresented in differentially methylated *Arabidopsis thaliana* genes in different sequence contexts (CpG, CHG, CHH).

We did similar analysis on Reactome pathways data [34] to identify pathways in which differentially methylated genes are involved (Figure 7). Using enrichment analysis, we found overrepresented pathways in different sequence contexts. Results with a significance of *p* < 0.05 after false discovery rate correction were taken to be significant. As in the case of gene ontology enrichment analysis, we observe that overrepresented features, which are common to all sequence contexts, are the most specific and related to the response to stress especially to action of heat shock proteins (HSP), translation elongation, and nonsense mediated decay (NMD), which serves as a major regulator of the unfolded protein response pathway [35].

**Figure 8:**
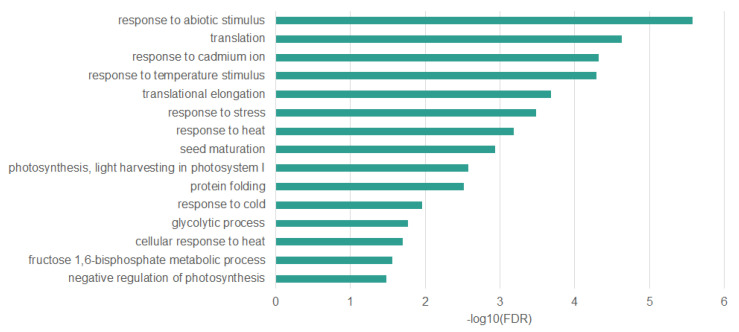
Biological processes overrepresented in *Arabidopsis thaliana* genes differentially methylated in all sequence contexts (CpG, CHG, CHH).

Many genes that we have identified as differentially methylated are associated with a response to stress (full list in Table S2). Especially interesting are those that have altered DNA methylation level in all sequence contexts, as they appear to be the most specifically related to the response to heat stress. We identified 45 genes with altered DNA methylation level in all sequence contexts. Ontology enrichment analysis of these genes (Figure 8) allowed to identify overrepresented biological processes such as response to heat (False Discovery Rate (FDR)—6.48 × 10^−4^; Fold Enrichment (FE)—15.85), protein folding (FDR—3.02 × 10^−3^; FE—16.49), translation (FDR—3.02 × 10^−3^; FE—11.71), and response to stress (FDR—3.30E-04; FE—3.47). All of these overrepresented terms are strictly associated with response to heat stress [7,31].

Among the genes associated with these overrepresented processes we can distinguish genes encoding heat shock proteins (Hsps) involved in refolding of proteins that form aggregates under heat stress. We found six differentially methylated genes encoding Hsps (HSP70, HSP70-1, HSP70-6, HSP81-2, HSP90-1, HSP101) that were differentially methylated in all sequence contexts (Table 1). Furthermore, we identified four Hsps that were differentially methylated in CHGs and CHHs (HSP70-5, HSP70-7, HSP90.3, MTHSP70-1) and seven that were differentially methylated only in CHHs (HSP70-3, HSP22.0, HSP70-2, HSP17.6, HSP17.7, HSP81-2, HSP90.7). However, in most cases, when we observed the change in DNA methylation level only in one or two contexts, we also observed changes in other contexts although not statistically significant with assumed criteria.

In addition to genes encoding heat shock proteins, we have also identified many other differentially methylated genes that are involved in different stress response signaling pathways. Example of such gene is LEA46 that encodes protein, which typically accumulates in response to low water availability conditions imposed during development or by external conditions like heat stress or drought [36]. Other genes associated with the response to water deprivation are POX1 and CIPK6.

**Figure 9:**
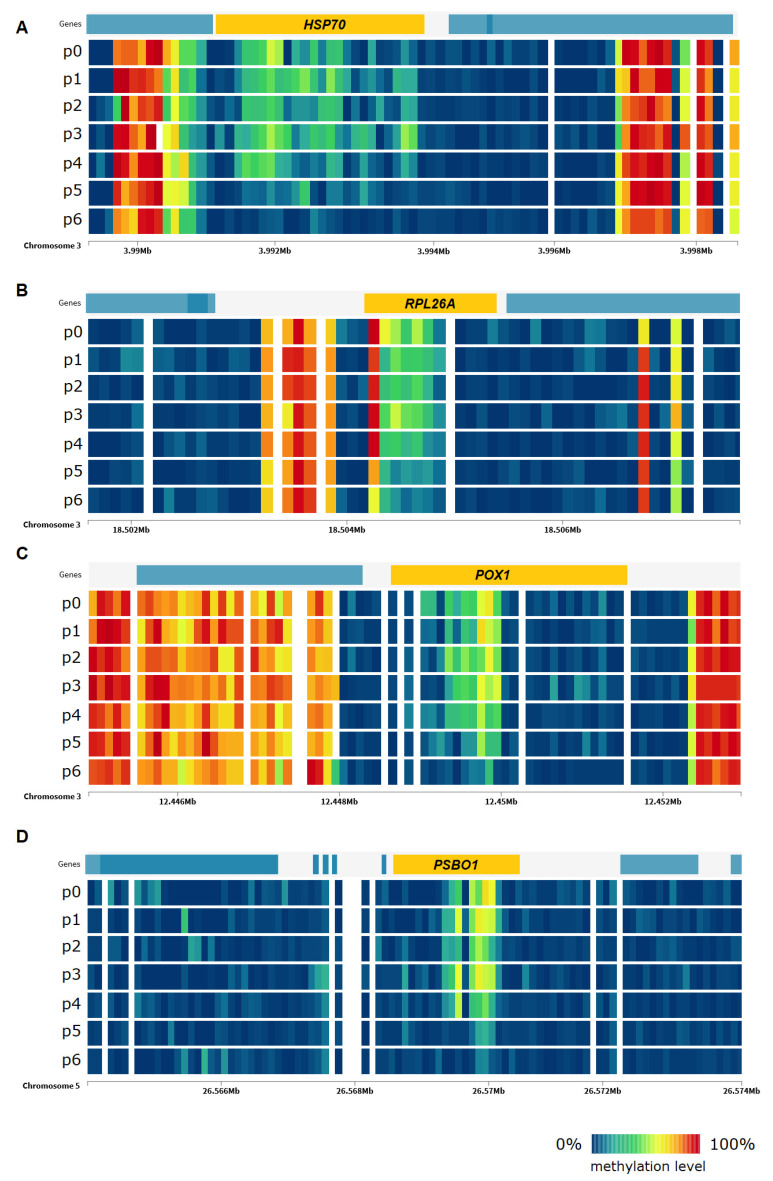
DNA methylation level in *Arabidopsis thaliana* genes ((**A**)—*HSP70*, (**B**)—*RPL26A*, (**C**)—*POX1*, (**D**)—*PSBO1*) in CpG context under heat stress in the subsequent time points of the experiment.

Another group of genes are those related to abscisic acid (ABA), which is a major phytohormone that plays an essential part in acting toward varied range of stresses [37] like heavy metal stress, drought, thermal or heat stress, high level of salinity, low temperature, and radiation stress. This category includes genes like GRDP1. As heat stress induces oxidative damage in plants [38], among differentially methylated genes there are also those associated with oxidation-reduction process and response to oxidative stress like FDH1 and PSBO1. Important group of genes are also those related to translation. Studies showed that regulation of translation elongation represents a major component of cellular stress responses [31]. Among these genes, RPL26A and RPL12A can be mentioned. Visualization of changes in DNA methylation level of selected stress-related genes under the influence of heat stress is presented in Figure 9.

## 3. Discussion

Recent studies have shown that epigenetic mechanisms are crucial in plant adaptation to stressful conditions. One of the most important epigenetic phenomena is DNA methylation. Studies show that the level of DNA methylation changes under the influence of various stresses such as salt stress [50,51], temperature stress [52,53,54], and drought [25,55,56,57,58] in various plant species. However, many of these studies have not been conducted on a global scale, but rather with the use of techniques allowing to study only some parts of the genome, such as the MSAP (Methylation Sensitive Amplified Polymorphism) method, which allows checking the status of only those places in the genome which are recognized by methylation-sensitive restriction enzymes like *Hpa*II. In addition, there are few studies that show changes in the level of methylation at many time points, also after the stress treatment discontinuation and recovery to optimal conditions. Due to the fact that the role of DNA methylation can be crucial in plant response to stress, understanding how it comes to changes in DNA methylation under stressful conditions will allow a better understanding of the mechanisms of plant response to environmental factors.

In the present study, whole-genome bisulfite sequencing was performed on DNA extracted from leaves of wild-type *A. thaliana* seedlings subjected to high temperature (42 °C). Analysis was performed at seven time points including control conditions, heat stress, and return to control conditions. The first stage of our analysis was to check the direction of changes in the level of DNA methylation. Results that we obtained show a reduction in the level of methylation under the influence of heat stress. We observed a gradual reduction in the level of methylation during the experiment time-course, with the largest differences detected by comparing the initial and last points of the analysis. The results were consistent for all sequence contexts (CpG, CHG, CHH). Analysis of changes taking place in subsequent time points showed that changes in the level of methylation occurred mainly after the end of heat stress and transfer to control conditions. We found very few hypo- and hypermethylation events during the first hours of the experiment. The results may suggest a delayed response to stress, which only becomes apparent after a few hours after the end of stress.

According to literature it seems that in most cases, a decrease in the level of methylation under heat stress is observed, however there are also studies showing the opposite results. Reduction in the global DNA methylation level under heat stress was observed in *Populus simonii* leaves [55], *Brassica napus* microspores [59], and *Gossypium hirsutum* anthers [60]. There is also study that showed that exposure of *A. thaliana* plants to heat stress increased global genome methylation and higher tolerance to stress in the untreated progeny, however authors also observed many hypomethylated loci [61]. Only the studies on *Brassica napus* microspores was conducted on a global scale using whole-genome bisulfite sequencing similarly to our studies.

Taking into account the time of occurrence of changes in the level of DNA methylation and the direction of these changes, we can conclude that for most differentially methylated genes DNA methylation level does not change significantly during the heat stress treatment, but decreases after its discontinuation in the last points of the experiment. However, we identified a small group of genes that methylation increases during stress and decreases after its discontinuation and transfer to control conditions. Interestingly, we did not identify any gene in which methylation level increases under stress and does not decrease after treatment discontinuation and transfer to control conditions. Decrease in the level of DNA methylation after stopping stress occurred in every case where we observed changes in the level of DNA methylation. The results show a great advantage of conducting the experiment at many time points, also after treatment discontinuation, as this mechanism would not be shown if the experiment was stopped just after treatment discontinuation. There are very few studies that also include analysis of samples collected after stopping stress treatment.

Ontology enrichment analysis and pathway enrichment analysis indicated that genes with differentially methylated regions are mainly involved in stress response, especially those with changed methylation level in all sequence contexts. Our analyses show that under the influence of heat stress, the level of DNA methylation is reduced and it refers to a specific genes, largely related to the response to stress. These results suggest the occurrence of active demethylation phenomenon rather than passive demethylation. Active DNA demethylation is locus specific and involves the enzymatic removal of methylated cytosine. In plants, this process is initiated by a family of DNA glycosylases including Demeter (DME), Repressor of silencing 1 (ROS1), Demeter-like 2 (DML2), and Demeter-like 3 (DML3) that can excise 5-methylcytosines in all sequence contexts [62]. In turn, passive DNA demethylation is not locus-specific and is caused by lack of DNA methyltransferase activity or shortage of a methyl donor following DNA replication.

Heat stress response in plants is controlled by various mechanisms, which are regulated by a set of genes with a specific function. Heat stress factors (Hsfs) and heat shock proteins (Hsps) play a central role in the heat stress and acquired thermotolerance in plants. Hsfs serve as the terminal component of signal transduction and mediates the expression of Hsps [63]. Hsp families, including Hsp100, Hsp90, Hsp70, Hsp60, and small Hsps (sHsps), are essential for normal growth and development in plants [64]. Hsps general role is to act as molecular chaperones regulating the folding and accumulation of proteins as well as localization and degradation [65,66,67]. These proteins, as chaperones, prevent the irreversible aggregation of other proteins and participate in refolding proteins during heat stress conditions. Each group of these Hsps has a unique mechanism [7]. In our studies we found over a dozen differentially methylated genes encoding Hsps.

Heat stress produces a massive global decrease in translation [68,69,70,71]. The global decrease in translation after heat stress is thought to be a consequence of ribosome pausing that is probably caused by the downregulation of HSP70 in the ribosomes after heat stress. Abnormal folding of the nascent protein under heat stress causes the ribosomes to pause, leading to mRNA decay [31]. Regulation of translation elongation in general, and by chaperones in particular, represents a major component of cellular stress responses. In our studies, using gene ontology enrichment and pathways enrichment we identified overrepresented processes associated with translation and involved in nonsense mediated decay (NMD) which serves as a major regulator of the unfolded protein response pathway [72]. Among differentially methylated genes we can mention *60S RIBOSOMAL PROTEIN L4-1* (*RPL4A*), *RPL8A*, *RPL12A*, *RPL26A*, *RPL27B*, *RPL27C.*

In addition to the genes typically associated with the response to heat stress we also found a lot of differentially methylated genes with documented role in response to various stresses, like osmotic stress, cold, metal ion, and high light intensity. Some of these genes have a proven role in responding to specific stress, which does not exclude the possibility of participating also in response to other stress, including heat stress. An example of such gene is fructose-bisphosphate aldolase 6 (FBA6) that has documented role in response to salt and cadmium ion stress [48,73], but its expression drastically increases under heat stress [74,75], which can suggest a role also in heat stress response. Another example is *CFBP1* that has a documented role in response to cold stress [76] or *POX1* with documented role in response to oxidative stress [77]. We also identified differentially methylated genes associated with response to water deprivation, which can occur as a result of prolonged heat stress. An example might be a gene LEA46 [36].

Although the role of gene body DNA methylation in gene expression is not fully understood and we do not know what effect DNA methylation has on the activity of genes, it seems that such a high representation of Hsps and other stress-related genes among differentially methylated genes under heat stress is not accidental. It is probable that DNA methylation participates in the regulation of their activity and in this way, it takes part in plant response to stress. Recent studies suggest mechanism in which DNA methylation may not be directly involved in the rapid induction of changes in the level of gene expression during the early stages of stress, however it can act as a marker within chromatin that preserves its condensation state and genes expression level established under stress. It is known that during replication, DNA methylation is an important marker that informs about the status of chromatin condensation and mainly thanks to this mechanism it is possible to reconstruct the entire nucleosomal complex at a given locus [17,78]. Similarly, under stress conditions, DNA methylation can be a factor that protects and fixes all elements of the complex responsible for the reorganization of the chromatin state [79,80,81].

Compared to the research on other species, we have identified few changes in DNA methylation level, however this is due to the specificity of the *A. thaliana* genome, which is small in size with low repetitive element content [82]. Observed changes in DNA methylation level occur in very specific places of the genome, in particular in the coding parts of genes. Due to the fact that a very small part of the *A. thaliana* promoters is methylated, we found very few differentially methylated promoters.

To conclude, we showed, at base-resolution level, that DNA methylation level changes under the influence of heat stress. We observed reduction in DNA methylation level several hours after treating plants with heat stress. This reduction in DNA methylation level occurs in specific genes, largely those associated with the stress response. Among differentially methylated genes we observed several heat shock proteins, genes involved in regulation of translational elongation which represents a major component of cellular stress responses and many genes with documented role in response to various abiotic stresses like salt, cold, metal ion and high light intensity. Considering the fact that changes in the level of DNA methylation were related to specific genes involved in the response to stress we assume that we observe active demethylation phenomenon induced in response to heat stress.

## 4. Materials and Methods

### 4.1. Plant Material, Growth, and Stress Conditions

Seeds of *A. thaliana* ecotype Columbia were sown on Jiffy pots (Jiffy, Moerdijk, Netherlands) and stratified in the darkness at 4 °C for 48 h. After stratification, seeds were transferred to the growth chamber (16 h photoperiod, photosynthetic photon flux density (PPFD) of 100 µmol m^−2^s^−1^ and temperature 21 °C) and regularly watered. After 21 days, seedlings were subjected to heat stress (42 °C) for 24 h. Fourth, fifth, and sixth leaves from control plants were collected just before temperature increase, while leaves from treated plants were harvested in 3 time points—after 6, 12, and 24 h. During stress treatment plants were not watered. After 24 h of heat stress, standard temperature conditions were restored together with watering. Leaves from recovering plants were collected after another 6, 12, and 24 h. The tissue samples were frozen immediately in liquid nitrogen and stored at −80 °C until DNA isolation.

### 4.2. Nucleic Acids Isolation

Leaves tissue that was harvested in three biological replicates at each of the seven time points described above was used for DNA isolation. Genomic DNA was extracted using the micro C-TAB procedure [83]. The purity of the DNA samples was determined using a NanoDrop ND-1000 spectrophotometer (Thermo Scientific, Waltham, MA, USA), whereas the integrity was evaluated using agarose gel electrophoresis. An exact estimation of genomic DNA concentration was conducted using fluorimetry with dsDNA specific dye and QuantiFluor Single Tube Fluorometer instrument (Promega, Madison, WI, USA).

### 4.3. WGBS Library Preparation and Sequencing

One microgram of genomic DNA was fragmented in 50 µL of 0.5× Tris-EDTA buffer using Bioruptor Plus sonication device (Diagenode, Liège, Belgium) under 10 cycles of 30 s ON and 90 s OFF power conditions. Then, fragmented DNA was purified with 1.8× Agencourt AMPure XP (Beckman Coulter, Brea, CA, USA) and eluted in 40 µL of ddH_2_O. Whole genome bisulfite sequencing libraries were prepared using the NEXTflex Bisulfite-Seq Kit (BIOO Scientific, Austin, TX, USA) according to the manufacturer’s instructions with minor changes. Briefly, genomic DNA fragments were subjected to end repair, clean-up, and adenylation. Next, barcoded adapters were ligated. The ligation products were purified and size-selected with Agencourt AMPure XP (Beckman Coulter, Brea, CA, USA). Prepared adapter-ligated fragments were subjected to bisulfite conversion with the EZ DNA Methylation-Lightning Kit (Zymo Research, Irvine, CA, USA) according to manufacturer’s protocol, and eluted in 17 µL of M-Elution buffer. Five microliters of converted DNA was amplified in 50 µL volume of PCR reaction (1.25 U of EpiMark Hot Start Taq DNA Polymerase, 1× dedicated buffer, 200 µM dNTPs and 2 µL of NEXTflex Primer Mix) under 1 min of initial denaturation in 95 °C, followed by 18 cycles of 30 s of denaturation in 95 °C 30 s of annealing in 65 °C, and 45 s of elongation in 68 °C, then 5 min of final elongation in 68 °C was applied. Amplicons were purified twice with Agencourt AMPure XP (Beckman Coulter, Brea, CA, USA). The quality of the prepared WGBS libraries was analyzed using Agilent Bioanalyzer and the Agilent High Sensitivity DNA Kit (Agilent Technologies, Santa Clara, CA, USA). Their concentrations were quantified using the Qubit Fluorometer (Thermo Fisher Scientific, Waltham, MA, USA). For cluster generation, WGBS libraries were pooled with equimolar concentrations, and sequenced using the Illumina HiSeq 4000 system (Illumina, San Diego, CA, USA) in 2× 76 cycles of paired-end (PE) mode with 11 barcoded samples per lane.

### 4.4. Bioinformatics Analysis

Raw sequence reads were trimmed to remove poor quality calls using BBduk [84]. Remaining sequences were mapped to the *A. thaliana* TAIR10 genome using Bismark [85]. Duplicated reads were removed. Percentage methylation and read coverage at each CpG, CHG and CHH site was determined by running the appropriate Bismark scripts. In order to exclude unreliable data, we incorporated a data filtering step. Data were filtered on the basis of coverage level. Only cytosines covered by at least 3 reads remained after filtration and were used for further calculations. Mt and Pt chromosomes were excluded from the analysis. Annotation of cytosines and average methylation of genes and genomic regions (exons, introns, UTRs and promoters defined as regions up to 1000 bp long upstream from the transcription start site) was determined using in-house scripts. Differentially methylated cytosines in each context were identified using Radmeth [30] while differentially methylated genes were identified with Metilene [86]. Differentially methylated genes were identified using predefined regions including intron, exons, and UTRs of each *A. thaliana* gene specified in a BED file. Gene ontology enrichment and pathway analysis was performed with Panther [87]. The raw and processed data were deposited into the GEO repository under the accession number GSE139941.

## Figures and Tables

**Table 1:** *Arabidopsis thaliana* genes associated with response to stress differentially methylated in gene body under heat stress in all three sequence contexts (CpG, CHG, CHH).

Gene Function	Gene ID	Gene Name	Gene Description	References
Protein folding, response to heat	AT3G12580	*HSP70*	*Heat shock protein 70*	[39]
	AT5G02500	*HSP70-1*	*Heat shock protein 70-1*	[40]
	AT4G24280	*HSP70-6*	*Heat shock protein 70-6*	[41]
	AT5G56030	*HSP81-2*	*Heat shock protein 81-2*	[42]
	AT5G52640	*HSP90-1*	*Heat shock protein 90-1*	[43]
	AT1G74310	*HSP101*	*Heat shock protein 101*	[44]
Regulation of translation	AT3G49910	*RPL26A*	*60S ribosomal protein L26-1*	[41]
	AT2G37190	*RPL12A*	*60S ribosomal protein L12-1*	[41]
Response to water deprivation	AT5G06760	*LEA46*	*Late embryogenesis abundant protein 46*	[36]
	AT3G30775	*POX1*	*Proline dehydrogenase 1*	[45]
	AT4G30960	*CIPK6*	*CBL-interacting serine/threonine-protein kinase 6*	[46]
ABA signaling pathway	AT2G22660	*GRDP1*	*Glycine-rich domain-containing protein 1*	[47]
Oxidation-reduction process	AT5G14780	*FDH1*	*Formate dehydrogenase*	[48]
	AT5G66570	*PSBO1*	*Oxygen-evolving enhancer protein 1-1*	[49]

## Data Availability

Publicly available datasets were analyzed in this study. This data can be found here: https://www.ncbi.nlm.nih.gov/geo/query/acc.cgi?acc=GSE139941.

## Supplementary Materials

Supplementary materials can be found at https://www.mdpi.com/1422-0067/22/4/1555/s1.

## Abbreviations

DMC	differentially methylated cytosine
HSP	heat shock protein
