# Supplementary material for: DNA Demethylation in Response to Heat Stress in Arabidopsis thaliana
Source: Int J Mol Sci. 2021 Feb 4;22(4):1555. doi: 10.3390/ijms22041555 (PMC7913789; doi:10.3390/ijms22041555)
Supplement: Supplementary file 1 [file ijms-22-01555-s001.zip › Table S2.pdf]

Table S2. Differentially methylated genes associated with response to environmental stress. The plus and minus signs indicate whether changes in the level of DNA methylation have been identified in a given context.

**Gene id**	**Gene symbol**	**CpG**	**CHG**	**CHH**
AT1G74310	*HEAT SHOCK PROTEIN 101 (HSP101)*	+	+	+
AT2G22660	*GLYCINE-RICH DOMAIN PROTEIN1 (ATGRDP1)*	+	+	+
AT2G36460	*FRUCTOSE-BISPHOSPHATE ALDOLASE 6 (FBA6)*	+	+	+
AT2G37190	*60S RIBOSOMAL PROTEIN L12-1 (RPL12A)*	+	+	+
AT3G12580	*HEAT SHOCK PROTEIN 70 (HSP70)*	+	+	+
AT3G22960	*PKP-ALPHA*	+	+	+
AT3G30775	*EARLY RESPONSIVE to DEHYDRATION 5 (ERD5)*	+	+	+
AT3G49910	*60S RIBOSOMAL PROTEIN L26-1 (RPL26A)*	+	+	+
AT3G54050	*HIGH CYCLIC ELECTRON FLOW 1 (HCEF1)*	+	+	+
AT4G24280	*CHLOROPLAST HEAT SHOCK PROTEIN 70-1 (CPHSC70-1)*	+	+	+
AT4G30960	*SOS3-INTERACTING PROTEIN 3 (SIP3)*	+	+	+
AT5G02500	*HEAT SHOCK COGNATE PROTEIN 70-1 (HSC70-1)*	+	+	+
AT5G06760	*LATE EMBRYOGENESIS ABUNDANT 4-5 (LEA4-5)*	+	+	+
AT5G14780	*FORMATE DEHYDROGENASE (FDH)*	+	+	+
AT5G52640	*HEAT SHOCK PROTEIN 90.1 (HSP90.1)*	+	+	+
AT5G52920	*PLASTIDIC PYRUVATE KINASE BETA SUBUNIT 1 (PKP-BETA1)*	+	+	+
AT5G56030	*HEAT SHOCK PROTEIN 81-2 (HSP81-2)*	+	+	+
AT5G64570	*BETA-D-XYLOSIDASE 4 (XYL4)*	+	+	+
AT5G66570	*PS II OXYGEN-EVOLVING COMPLEX 1 (PSBO1)*	+	+	+
AT4G38740	*ROTAMASE CYP 1 (ROC1)*	+	+	+
AT1G13740	*ABI FIVE BINDING PROTEIN 2 (AFP2)*	+	-	+
AT1G20440	*COLD-REGULATED 47 (COR47)*	+	-	+
AT1G23740	*ALKENAL/ONE OXIDOREDUCTASE (AOR)*	+	-	+
AT1G32560	*LATE EMBRYOGENESIS ABUNDANT 4-1 (ATLEA4-1)*	+	-	+
AT1G48130	*1-CYSTEINE PEROXIREDOXIN 1 (PER1)*	+	-	+
AT2G07698	*ATPASE, F1 COMPLEX, ALPHA SUBUNIT PROTEIN*	+	-	+
AT3G16460	*JACALIN-RELATED LECTIN 34 (JAL34)*	+	-	+
AT3G27660	*OLEOSIN 4 (OLEO4)*	+	-	+
AT3G56860	*UBP1-ASSOCIATED PROTEIN 2A (UBA2A)*	+	+	-
AT4G10250	*ATHSP22.0*	+	-	+
AT5G48570	*ROF2*	+	+	-
AT1G04120	*ATP-BINDING CASSETTE C5 (ABCC5)*	-	+	+
AT1G05850	*POM-POM1 (POM1)*	-	+	+
AT1G20450	*EARLY RESPONSIVE to DEHYDRATION 10 (ERD10)*	-	+	+
AT1G35720	*ANNEXIN 1 (ANNAT1)*	-	+	+
AT1G50010	*TUBULIN ALPHA-2 CHAIN (TUA2)*	-	+	+
AT1G52690	*LATE EMBRYOGENESIS ABUNDANT 7 (LEA7)*	-	+	+
AT1G56070	*LOW EXPRESSION OF OSMOTICALLY RESPONSIVE GENES 1 (LOS1)*	-	+	+
AT1G61520	*PHOTOSYSTEM i LIGHT HARVESTING COMPLEX GENE 3 (LHCA3)*	-	+	+
AT2G01140	*PIGMENT DEFECTIVE 345 (PDE345)*	-	+	+
AT2G21490	*DEHYDRIN LEA (LEA)*	-	+	+

AT2G28290	*SPLAYED (SYD)*	-	+	+
AT2G39460	*RIBOSOMAL PROTEIN L23AA (RPL23AA)*	-	+	+
AT2G39770	*CYTOKINESIS DEFECTIVE 1 (CYT1)*	-	+	+
AT2G43710	*SUPPRESSOR OF SA INSENSITIVE 2 (SSI2)*	-	+	+
AT3G01570	*OLEOSIN 5*	-	+	+
AT3G02360	*6-PHOSPHOGLUCONATE DEHYDROGENASE 2 (PGD2)*	-	+	+
AT3G04120	*GLYCERALDEHYDE-3-PHOSPHATE DEHYDROGENASE C SUBUNIT 1 (GAPC1)*	-	+	+
AT3G09200	*60S ACIDIC RIBOSOMAL PROTEIN P0-2 (RPP0B)*	-	+	+
AT3G09840	*CDC48A*	-	+	+
AT3G26650	*GLYCERALDEHYDE 3-PHOSPHATE DEHYDROGENASE A SUBUNIT (GAPA)*	-	+	+
AT3G52930	*FRUCTOSE-BISPHOSPHATE ALDOLASE 8 (FBA8)*	-	+	+
AT3G55980	*SALT-INDUCIBLE ZINC FINGER 1 (SZF1)*	-	+	+
AT4G14960	*TUBULIN ALPHA-6 (TUA6)*	-	+	+
AT4G25140	*OLEOSIN 1 (OLEO1)*	-	+	+
AT4G37870	*PHOSPHOENOLPYRUVATE CARBOXYKINASE 1 (PCK1)*	-	+	+
AT4G37910	*MITOCHONDRIAL HEAT SHOCK PROTEIN 70-1 (mtHsc70-1)*	-	+	+
AT5G07350	*TUDOR-SN PROTEIN 1 (TUDOR1)*	-	+	+
AT5G08590	*SNF1-RELATED PROTEIN KINASE 2.1 (SNRK2.1)*	-	+	+
AT5G09590	*MITOCHONDRIAL HSO70 2 (MTHSC70-2)*	-	+	+
AT5G12250	*BETA-6 TUBULIN (TUB6)*	-	+	+
AT5G17920	*METHIONINE SYNTHESIS 1 (ATMS1)*	-	+	+
AT5G20010	*RAS-RELATED NUCLEAR PROTEIN-1 (RAN-1)*	-	+	+
AT5G42020	*BIP2*	-	+	+
AT5G49910	*CHLOROPLAST HEAT SHOCK PROTEIN 70-2 (cpHsc70-2)*	-	+	+
AT5G56010	*HEAT SHOCK PROTEIN 81-3 (HSP81-3)*	-	+	+
AT5G60640	*PDI-LIKE 1-4 (PDIL1-4)*	-	+	+
AT5G61780	*TUDOR-SN PROTEIN 2 (TUDOR2)*	-	+	+
AT1G68530	*3-KETOACYL-COA SYNTHASE 6 (KCS6)*	+	-	-
AT1G78080	*RELATED to AP2 4 (RAP2.4)*	+	-	-
AT3G12630	*STRESS ASSOCIATED PROTEIN 5 (SAP5)*	+	-	-
AT5G49480	*CA2+-BINDING PROTEIN 1 (CP1)*	+	-	-
AT5G59220	*HIGHLY ABA-INDUCED PP2C GENE 1 (HAI1)*	+	-	-
AT5G64940	*ABC2 HOMOLOG 13 (ATH13)*	+	-	-
AT5G65430	*GENERAL REGULATORY FACTOR 8 (GRF8)*	+	-	-
AT5G67590	*FROSTBITE1 (FRO1)*	+	-	-
AT2G33770	*PHOSPHATE 2 (PHO2)*	-	+	-
AT4G32260	*PIGMENT DEFECTIVE 334 (PDE334)*	-	+	-
AT5G21010	*BTB-POZ AND MATH DOMAIN 5 (BPM5)*	-	+	-
AT5G26742	*EMBRYO DEFECTIVE 1138 (EMB1138)*	-	+	-
AT1G74840	*F25A4.19 PROTEIN*	-	-	+
AT1G51400	*F5D21_10*	-	-	+
AT3G09440	*HEAT SHOCK PROTEIN 70 (HSP70-3)*	-	-	+
AT2G27710	*RPP2B*	-	-	+
AT3G12260	*NADH DEHYDROGENASE [UBIQUINONE] 1 ALPHA SUBCOMPLEX SUBUNIT 6*	-	-	+

AT4G24275	*AT4G24275*	-	-	+
AT3G45930	*HISTONE H4*	-	-	+
AT2G37220	*RNA-BINDING PROTEIN CP29B*	-	-	+
AT5G08790	*ATAF2*	-	-	+
AT1G15690	*AVP1*	-	-	+
AT5G28540	*BIP1*	-	-	+
AT5G02490	*HSP70-2*	-	-	+
AT3G44110	*J3*	-	-	+
AT1G48050	*KU80*	-	-	+
AT5G47690	*PDS5A*	-	-	+
AT4G21960	*PRXR1*	-	-	+
AT3G09260	*PYK10*	-	-	+
AT1G11650	*RBP45B*	-	-	+
AT5G54770	*THI1*	-	-	+
AT5G12020	*17.6 KDA CLASS II HEAT SHOCK PROTEIN (HSP17.6II)*	-	-	+
AT2G26250	*3-KETOACYL-COA SYNTHASE 10 (KCS10)*	-	-	+
AT2G19590	*ACC OXIDASE 1 (ACO1)*	-	-	+
AT3G44880	*ACCELERATED CELL DEATH 1 (ACD1)*	-	-	+
AT1G36160	*ACETYL-COA CARBOXYLASE 1 (ACC1)*	-	-	+
AT2G05710	*ACONITASE 3 (ACO3)*	-	-	+
AT5G09810	*ACTIN 7 (ACT7)*	-	-	+
AT3G48990	*ACYL-ACTIVATING ENZYME 3 (AAE3)*	-	-	+
AT1G77120	*ALCOHOL DEHYDROGENASE 1 (ADH1)*	-	-	+
AT1G73680	*ALPHA DIOXYGENASE (ALPHA DOX2)*	-	-	+
AT3G46970	*ALPHA-GLUCAN PHOSPHORYLASE 2 (PHS2)*	-	-	+
AT5G55240	*ARABIDOPSIS THALIANA PEROXYGENASE 2 (ATPXG2)*	-	-	+
AT4G34710	*ARGININE DECARBOXYLASE 2 (ADC2)*	-	-	+
AT2G27040	*ARGONAUTE 4 (AGO4)*	-	-	+
AT5G67480	*BTB AND TAZ DOMAIN PROTEIN 4 (BT4)*	-	-	+
AT1G56340	*CALRETICULIN 1A (CRT1a)*	-	-	+
AT1G29900	*CARBAMOYL PHOSPHATE SYNTHETASE B (CARB)*	-	-	+
AT1G09770	*CELL DIVISION CYCLE 5 (CDC5)*	-	-	+
AT1G02730	*CELLULOSE SYNTHASE-LIKE D5 (CSLD5)*	-	-	+
AT5G46210	*CULLIN4 (CUL4)*	-	-	+
AT1G22450	*CYTOCHROME C OXIDASE 6B (COX6B)*	-	-	+
AT3G53280	*CYTOCHROME P450 71B5 (CYP71B5)*	-	-	+
AT4G39800	*D-MYO-INOSITOL 3-PHOSPHATE SYNTHASE 1 (MIPS1)*	-	-	+
AT1G75270	*DEHYDROASCORBATE REDUCTASE 2 (DHAR2)*	-	-	+
AT2G36490	*DEMETER-LIKE 1 (DML1)*	-	-	+
AT5G20320	*DICER-LIKE 4 (DCL4)*	-	-	+
AT1G56280	*DROUGHT-INDUCED 19 (DI19)*	-	-	+
AT3G22840	*EARLY LIGHT-INDUCABLE PROTEIN (ELIP1)*	-	-	+
AT4G14690	*EARLY LIGHT-INDUCIBLE PROTEIN 2 (ELIP2)*	-	-	+
AT2G41430	*EARLY RESPONSIVE to DEHYDRATION 15 (ERD15)*	-	-	+
AT5G37510	*EMBRYO DEFECTIVE 1467 (EMB1467)*	-	-	+
AT1G18260	*EMS-MUTAGENIZED BRI1 SUPPRESSOR 5 (EBS5)*	-	-	+
AT5G43060	*ESPONSIVE to DEHYDRATION 21B (RD21B)*	-	-	+

AT3G20770	*ETHYLENE-INSENSITIVE3 (EIN3)*	-	-	+
AT1G74960	*FATTY ACID BIOSYNTHESIS 1 (FAB1)*	-	-	+
AT3G51550	*FERONIA (FER)*	-	-	+
AT5G66190	*FERREDOXIN-NADP(+)-OXIDOREDUCTASE 1 (FNR1)*	-	-	+
AT4G11600	*GLUTATHIONE PEROXIDASE 6 (GPX6)*	-	-	+
AT2G30860	*GLUTATHIONE S-TRANSFERASE PHI 9 (GSTF9)*	-	-	+
AT1G42970	*GLYCERALDEHYDE-3-PHOSPHATE DEHYDROGENASE B SUBUNIT (GAPB)*	-	-	+
AT5G49720	*GLYCOSYL HYDROLASE 9A1 (GH9A1)*	-	-	+
AT2G18960	*H(+)-ATPASE 1 (HA1)*	-	-	+
AT5G12030	*HEAT SHOCK PROTEIN 17.6A (HSP17.6A)*	-	-	+
AT3G23990	*HEAT SHOCK PROTEIN 60 (HSP60)*	-	-	+
AT1G52740	*HISTONE H2A PROTEIN 9 (HTA9)*	-	-	+
AT2G44060	*LATE EMBRYOGENESIS ABUNDANT 26 (LEA26)*	-	-	+
AT5G54270	*LIGHT-HARVESTING CHLOROPHYLL B-BINDING PROTEIN 3 (LHCB3)*	-	-	+
AT3G47470	*LIGHT-HARVESTING CHLOROPHYLL-PROTEIN COMPLEX i SUBUNIT A4 (LHCA4)*	-	-	+
AT2G36530	*LOW EXPRESSION OF OSMOTICALLY RESPONSIVE GENES 2 (LOS2)*	-	-	+
AT3G17390	*METHIONINE OVER-ACCUMULATOR 3 (MTO3)*	-	-	+
AT4G21860	*METHIONINE SULFOXIDE REDUCTASE B 2 (MSRB2)*	-	-	+
AT3G24500	*MULTIPROTEIN BRIDGING FACTOR 1C (MBF1C)*	-	-	+
AT5G67300	*MYB DOMAIN PROTEIN R1 (MYBR1)*	-	-	+
AT2G22240	*MYO-INOSITOL-1-PHOSPHATE SYNTHASE 2 (MIPS2)*	-	-	+
AT5G13780	*NAA10 (NAA10)*	-	-	+
AT1G07380	*NEUTRAL CERAMIDASE 1 (ATNCER1)*	-	-	+
AT5G40420	*OLEOSIN 2 (OLEO2)*	-	-	+
AT2G28900	*OUTER PLASTID ENVELOPE PROTEIN 16-1 (OEP16-1)*	-	-	+
AT4G33220	*PECTIN METHYLESTERASE 44 (PME44)*	-	-	+
AT3G26060	*PEROXIREDOXIN Q (PRXQ)*	-	-	+
AT2G33150	*PEROXISOMAL 3-KETOACYL-COA THIOLASE 3 (PKT3)*	-	-	+
AT2G37040	*PHE AMMONIA LYASE 1 (pal1)*	-	-	+
AT5G23630	*PHOSPHATE DEFICIENCY RESPONSE 2 (PDR2)*	-	-	+
AT1G53310	*PHOSPHOENOLPYRUVATE CARBOXYLASE 1 (PPC1)*	-	-	+
AT1G79550	*PHOSPHOGLYCERATE KINASE (PGK)*	-	-	+
AT3G12780	*PHOSPHOGLYCERATE KINASE 1 (PGK1)*	-	-	+
AT2G46500	*PHOSPHOINOSITIDE 4-KINASE GAMMA 4 (PI4K GAMMA 4)*	-	-	+
AT1G32060	*PHOSPHORIBULOKINASE (PRK)*	-	-	+
AT3G54890	*PHOTOSYSTEM i LIGHT HARVESTING COMPLEX GENE 1 (LHCA1)*	-	-	+
AT3G61470	*PHOTOSYSTEM i LIGHT HARVESTING COMPLEX GENE 2 (LHCA2)*	-	-	+
AT1G06680	*PHOTOSYSTEM II SUBUNIT P-1 (PSBP-1)*	-	-	+
AT4G00430	*PLASMA MEMBRANE INTRINSIC PROTEIN 1;4 (PIP1;4)*	-	-	+
AT1G01620	*PLASMA MEMBRANE INTRINSIC PROTEIN 1C (PIP1C)*	-	-	+
AT4G35100	*PLASMA MEMBRANE INTRINSIC PROTEIN 3 (PIP3)*	-	-	+

AT2G29570	*PROLIFERATING CELL NUCLEAR ANTIGEN 2 (PCNA2)*	-	-	+
AT3G24550	*PROLINE-RICH EXTENSIN-LIKE RECEPTOR KINASE 1 (PERK1)*	-	-	+
AT3G11410	*PROTEIN PHOSPHATASE 2CA (PP2CA)*	-	-	+
AT1G49480	*RELATED to VERNALIZATION1 1 (RTV1)*	-	-	+
AT3G55280	*RIBOSOMAL PROTEIN L23AB (RPL23AB)*	-	-	+
AT1G67090	*RIBULOSE BISPHOSPHATE CARBOXYLASE SMALL CHAIN 1A (RBCS1A)*	-	-	+
AT1G25490	*ROOTS CURL IN NPA (RCN1)*	-	-	+
AT3G62030	*ROTAMASE CYP 4 (ROC4)*	-	-	+
AT4G24190	*SHEPHERD (SHD)*	-	-	+
AT5G62520	*SIMILAR to RCD ONE 5 (SRO5)*	-	-	+
AT5G16270	*SISTER CHROMATID COHESION 1 PROTEIN 4 (SYN4)*	-	-	+
AT5G49190	*SUCROSE SYNTHASE 2 (SUS2)*	-	-	+
AT5G23570	*SUPPRESSOR OF GENE SILENCING 3 (SGS3)*	-	-	+
AT3G11820	*SYNTAXIN OF PLANTS 121 (SYP121)*	-	-	+
AT5G58070	*TEMPERATURE-INDUCED LIPOCALIN (TIL)*	-	-	+
AT5G57560	*TOUCH 4 (TCH4)*	-	-	+
AT3G60750	*TRANSKETOLASE 1 (TKL1)*	-	-	+
AT5G13930	*TRANSPARENT TESTA 4 (TT4)*	-	-	+
AT3G55440	*TRIOSEPHOSPHATE ISOMERASE (TPI)*	-	-	+
AT5G54810	*TRYPTOPHAN SYNTHASE BETA-SUBUNIT 1 (TSB1)*	-	-	+
AT1G04820	*TUBULIN ALPHA-4 CHAIN (TUA4)*	-	-	+
AT5G62690	*TUBULIN BETA CHAIN 2 (TUB2)*	-	-	+
AT5G62700	*TUBULIN BETA CHAIN 3 (TUB3)*	-	-	+
AT2G29550	*TUBULIN BETA-7 CHAIN (TUB7)*	-	-	+
AT5G06460	*UBIQUITIN ACTIVATING ENZYME 2 (UBA 2)*	-	-	+
AT4G24560	*UBIQUITIN-SPECIFIC PROTEASE 16 (UBP16)*	-	-	+
AT2G47470	*UNFERTILIZED EMBRYO SAC 5 (UNE5)*	-	-	+
AT1G28520	*VASCULAR PLANT ONE ZINC FINGER PROTEIN (VOZ1)*	-	-	+
